# Bridging the gap: a genetically validated avian chorioallantoic membrane platform for investigation of spontaneous circulating tumor cells

**DOI:** 10.1002/1878-0261.70336

**Published:** 2026-09-18

**Authors:** Dennis Roth, Rui P. L. Neves, Julia Geheb, Christian Ballmeyer, Nathalie Gaßmann, Kiki C. Andree, Monica Sudarsanam, Christiane Driemel, Celine Örencik, Andrè Franken, Eslam Elmaghraby, Sven H. Loosen, Roderburg Christoph, Sebastian Schoelch, Hans Neubauer, Wolfram T. Knoefel, Nikolas H. Stoecklein, Georg Fluegen

**Affiliations:** ^1^ Department of Surgery (A) Heinrich‐Heine‐University and University Hospital Duesseldorf Germany; ^2^ Liquid Biopsy Center Duesseldorf Center for Integrated Oncology Aachen Bonn, Cologne Germany; ^3^ VyCAP B.V Enschede The Netherlands; ^4^ Department of Obstetrics and Gynecology University Hospital and Medical Faculty of the Heinrich‐Heine‐University Duesseldorf Germany; ^5^ Department of Gastroenterology, Hepatology and Infectious Diseases University Hospital Duesseldorf, Medical Faculty of Heinrich Heine University Duesseldorf Germany; ^6^ JCCU Translational Surgical Oncology (A430) German Cancer Research Center (DKFZ) Heidelberg Germany; ^7^ DKFZ‐Hector Cancer Institute, Medical Faculty Mannheim Heidelberg University Mannheim Germany; ^8^ Department of Surgery Universitätsmedizin Mannheim, Medical Faculty Mannheim, Heidelberg University Mannheim; ^9^ Department of General, Visceral, Vascular and Transplantation Surgery University Hospital Essen Germany

**Keywords:** 3R methods, chorioallantoic membrane (CAM), circulating tumor cells (CTCs), liquid biopsy, metastasis, single‐cell genomics

## Abstract

Circulating tumor cells (CTCs) are clinically relevant markers of metastasis and prognosis in gastrointestinal (GI) cancers. However, their extreme rarity and the limitations of current *in vivo* models severely restrict mechanistic studies of tumor cell dissemination. Here, we demonstrate for the first time that the avian chorioallantoic membrane (CAM) assay can serve as a biologically relevant and ethically advantageous 3R‐compliant (Replacement, Reduction, and Refinement) *in vivo* model for investigating spontaneous CTC generation. Six green fluorescent protein (GFP)‐labeled human colorectal, pancreatic, and cholangiocarcinoma cell lines were xenografted onto the CAM. Using a standardized workflow, we achieved reliable detection and isolation of GFP‐positive single CTCs from small‐volume CAM blood samples. Critically, we reproducibly confirmed the presence of spontaneously shed CAM‐derived CTCs independent of GFP expression using three established, clinically relevant CTC detection platforms. Subsequent single‐cell whole‐genome amplification enabled downstream short‐tandem repeat (STR) profiling to authenticate CTC identity, alongside detection of tumor‐specific point mutations and copy number alterations (CNAs). Isolated CTCs exhibited high genetic concordance with their parental cell lines, validating the technical robustness of our workflow and establishing the CAM assay as a versatile, scalable platform for investigating hematogenous dissemination and CTC biology outside of mammalian models. Consequently, the CAM assay opens new avenues for early mechanistic and pharmacological CTC research, including functional studies of patient‐derived material.

## Graphical abstract

We established the avian chorioallantoic membrane (CAM) assay as a scalable *in vivo* model for studying circulating tumor cells (CTCs). Human gastrointestinal tumors spontaneously released genetically validated CTCs that were detected across multiple platforms, demonstrating that the CAM model provides an accessible tool for investigating early cancer dissemination and developing antimetastatic therapies.

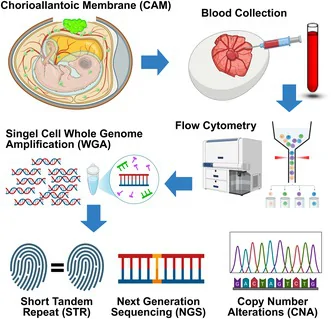

**Abbreviations**
BSA: bovine serum albumin
CAM: chorioallantoic membrane
CCA: cholangiocarcinoma
CNA: copy number alteration
CRC: colorectal cancer
CTC: circulating tumor cell
DAPI: 4′,6‐diamidino‐2‐phenylinodole
DLRS: derivative log ratio spread
DMEM: Dulbecco's Modified Eagle Medium
DNA: desoxyribonucleic acid
EDTA: ethylenediaminetetraacetic acid
EpCAM: epithelial cell adhesion molecule
FBS: fetal bovine serum
FDA: US Food and Drug Administration
FSC: forward scatter
GFP: green fluorescent protein
GI: gastrointestinal
GII: genome integrity index
HEK293T cells: human embryonic kidney 293T cells
HEPES: 4‐(2‐hydroxyethyl)‐1‐piperazineethanesulfonic acid
IQR: interquatile range
MACS: magnetic‐activated cell sorting
NGS: next‐generation sequencing
PBS: phosphate buffer saline
PCR: polymerase chain reaction
PDAC: pancreatic ductal adenocarcinoma
PDO: patient‐derived organoid
QDNAseq: quantitative DNA sequencing analysis pipeline
RRID: research resource identifier
SOP: standard operating procedure
SPF: specific‐pathogen‐free
SSC: side scatter
STR: short‐tandem repeat
WGA: whole‐genome amplification

## 1 Introduction

Gastrointestinal (GI) cancers, including colorectal cancer (CRC), pancreatic ductal adenocarcinoma (PDAC), and cholangiocarcinoma (CCA), collectively contribute to approximately 15–17% of cancer‐related deaths worldwide [1]. While CRC treatment has advanced substantially, metastatic disease causes nearly 50% of deaths, with a metastatic 5‐year survival rate of only 15% [2]. PDAC and CCA outcomes are markedly worse, exhibiting persistently high mortality and limited therapies despite extensive research efforts [3, 4, 5].

In these malignancies, hematogenous dissemination constitutes a principal determinant of adverse prognosis. Circulating tumor cells (CTCs), shed from the primary tumor or metastases into the bloodstream, are critically implicated as mediators of metastatic spread. Numerous studies have established that CTC detection correlates with advanced tumor stage and reduced survival in CRC, PDAC, CCA, and other GI cancers [6, 7, 8, 9]. Nevertheless, despite their established clinical relevance, reliable and reproducible CTC detection remains challenging, particularly during early disease stages when their frequency in peripheral blood is exceedingly low [6, 10, 11, 12, 13, 14, 15].

To date, mechanistic studies of tumor cell dissemination and CTC biology have largely relied on complex murine *in vivo* models. While invaluable, these models are resource‐intensive, time‐consuming, and subject to ethical constraints, limiting their utility for high‐throughput or early‐stage exploratory research.

The avian chorioallantoic membrane (CAM) assay provides a compelling, ethically favorable alternative *in vivo* model, adhering to the 3R principles (Replacement, Reduction, Refinement). Its accessibility, scalability, and cost‐effectiveness, combined with a highly vascularized, immunodeficient environment, enable efficient human tumor xenografting and real‐time monitoring of tumor growth, angiogenesis, and metastasis [16, 17, 18, 19, 20, 21]. Previous research has demonstrated the ability of xenografted human tumor cell lines to access the CAM bloodflow and disseminate [22].

In this study, we establish the CAM model as a novel, biologically relevant platform for investigating systemic dissemination, specifically capturing single CTCs in circulation. Utilizing CRC, PDAC, and CCA cell line‐derived xenografts, we demonstrate the reproducible detection and, crucially, isolation of CTCs spontaneously shed by the human xenografts from avian blood. Through single‐cell whole‐genome amplification followed by short‐tandem repeat (STR), hotspot mutation detection, and copy number alteration (CNA) analyses, we confirm the human origin and genetic integrity of isolated CTCs.

Collectively, our findings establish the CAM assay as a highly tractable *in vivo* model for investigating early metastatic dissemination in GI cancers, thus providing a much‐needed intermediary platform bridging the gap between simplified *in vitro* systems and highly complex murine models.

## 2 Material and methods

### 2.1 Cell culture

CRC (HCT‐116, SW‐620), PDAC (CAPAN‐1, Mia‐PaCa2), CCA (HuCC‐T1, TFK‐1), and human embryonic kidney (HEK293T) cell lines were obtained from DSMZ (Braunschweig, Germany). All cell lines were maintained at 37 °C/5% CO_2_ with media supplemented as follows:

HCT‐116 (RRID:CVCL_0291): McCoy's 5A (Gibco, Waltham, MA, USA) + 10% fetal bovine serum (FBS) + 1% Penicillin/Streptomycin (both Sigma, St. Louis, MO, USA).

SW‐620 (RRID:CVCL_0547): Leibovitz L‐15 (Gibco, USA) + 10% FBS + 1% Penicillin/Streptomycin (both Sigma, USA).

CAPAN‐1 (RRID:CVCL_0237): RPMI‐1640 (PAN‐Biotech, Aidenbach, Germany) + 20% FBS + 1% Penicillin/Streptomycin (both Sigma, USA).

Mia‐PaCa2 (RRID:CVCL_0428): DMEM (4.5 g·L^−1^ glucose; Gibco, USA) + 10% FBS + 2.5% horse serum (PAN‐biotech, Germany) + 1% Penicillin/Streptomycin (both Sigma, USA).

HuCC‐T1 (RRID:CVCL_0324) and TFK‐1 (RRID:CVCL_2214): RPMI‐1640 (Gibco, USA) + 10% FBS + 1% Penicillin/Streptomycin.

HEK293T (RRID:CVCL_0063): DMEM + GlutaMAX™ (Gibco, USA) + 10% FBS.

All cells were passaged using 0.05% Trypsin (PAN‐Biotech, Germany) per standard protocols. All cell lines were routinely authenticated by STR profiling and regularly tested for mycoplasma contamination.

### 2.2 Production of lentiviral particles and cell transduction

Lentiviral particles were produced by transfecting HEK293T cells. Briefly, 5 × 10^6^ HEK293T cells were plated in 10‐cm dishes and incubated overnight at 37 °C. The following day, a transfection mixture (275 μL total volume) containing 5 μg pCMV‐VSV‐G (Addgene, Watertown, MA, USA; #8454), 15 μg pPAX2 (Addgene #12260), and 20 μg pGIPZ (#RHS4430‐99292606; Open Biosystems, Huntsville, AL, USA) in 2.5 mm *(4‐(2‐hydroxyethyl)‐1‐piperazineethanesulfonic acid)* HEPES was prepared. This solution was mixed with 275 μL of 2 m CaCl_2_, added dropwise to 550 μL 2× HEPES buffer, and incubated statically for 30 min. HEK293T culture medium was replaced with 5 mL serum‐free medium, followed by dropwise addition of 1 mL transfection mixture. After 5 h at 37 °C, medium was replaced with complete culture medium. Viral supernatant was harvested 48 h post‐transfection and stored at −80 °C. Target cancer cell lines were transduced by replacing their medium with 600 μL viral supernatant. Following 24‐h incubation (37 °C, 5% CO_2_), supernatant was removed and replaced with 2 mL fresh culture medium.

### 2.3 Chorioallantoic membrane model and blood collection

All experimental procedures involving avian embryos were conducted in strict accordance with institutional guidelines and established standard operating procedures (SOPs) for the care and use of laboratory animals. According to European Law, the CAM is no animal experiment. We have registered our CAM work with the LAVE NRW (LV22‐2025‐0032598). Specific‐pathogen‐free (SPF) fertilized chicken eggs were incubated at 37.5 °C and 48% relative humidity for 10 days in a rotating incubator (30‐min rotation intervals, Fig. S1, Panel 1). On embryonic day 10 (E10), eggs were candled to identify a zone adjacent to well‐vascularized CAM regions, avoiding major blood vessels (Fig. S1, Panels 2–3). Following shell disinfection, a hole was drilled through all membranes into the air sac at the blunt end of the egg (Fig. S1, Panel 3). A superficial incision was made in the marked shell area without membrane penetration (Fig. S1, Panel 4). Air was then aspirated from the air sac using a medium bulb to drop the CAM (Fig. S1, Panel 5). Finally, the window was opened at the marked site using a rotary tool (Fig. S1, Panels 6 and 7). To define the inoculation site, a sterile silicone ring (sterile screw‐cap seals of 2‐mL cryovials; neoLab Migge GmbH, Heidelberg, Germany) was placed onto the CAM surface. A suspension of 1 × 10^6^ cancer cells in 50 μL phosphate buffer saline (PBS) was then inoculated into the center of the ring. Following inoculation, the eggshell window was sealed with transparent adhesive tape (Tesa SE, Norderstedt, Germany) to prevent dehydration and contamination while maintaining a stable environment for the developing embryo, and a suspension of 1 × 10^6^ cancer cells in 50 μL PBS was inoculated onto the CAM within a sterile silicone ring.

Six human CRC, PDAC, and CCA cell lines (HCT‐116, SW‐620, CAPAN‐1, Mia‐PaCa2, HuCC‐T1, TFK‐1) were used. These cell lines were specifically selected to allow for pairwise comparisons within each cancer entity, thereby reducing interentity variability and enabling a more focused analysis of spontaneous CTC shedding across different tumor characteristics. This included comparisons between primary and metastatic origins (CRC: HCT‐116 vs. SW‐620), different degrees of differentiation and aggressiveness (PDAC: CAPAN‐1 vs. Mia‐PaCa2), as well as different anatomical subtypes and growth patterns (CCA: HuCC‐T1 vs. TFK‐1). All cell lines were stably transduced in‐house using lentiviral particles to constitutively express green fluorescent protein (GFP) (Methods S1). Eggs were incubated statically at 37 °C for an additional 7 days.

On E17, 100 μL of heparin (100 IU; Ratiopharm, Ulm, Germany) was injected beneath the CAM into the albumen 30 min prior to blood collection to prevent coagulation. Blood was collected from CAM vasculature using syringes (Disponed, Gelnhausen‐Hailer, Germany) equipped with sterile 26G needles (0.45 × 25 mm; B. Braun, Melsungen, Germany), prefilled with 122 μL of 0.5 m EDTA. The collected blood volume was recorded. Immediately following blood collection, the chicken embryos were humanely sacrificed by decapitation in accordance with ethical standards for avian embryo research. Subsequently, CAM tumors were harvested and weighed.

### 2.4 Blood preparation and flow cytometry cell sorting

Red blood cells in CAM blood were lysed using 20 mL cold (4 °C) ammonium chloride‐based lysis buffer (8.29 mg·mL^−1^ NH_4_Cl, 1 mg·mL^−1^ KHCO_3_, 0.0375 mg·mL^−1^ Na_2_EDTA) for 25 min on ice, vortexing every 5 min. Samples were centrifuged at 300× ***g*** for 5 min at room temperature, washed twice with 20 mL cold PBS, and pellets resuspended in 350 μL magnetic‐activated cell sorting (MACS) buffer (Miltenyi Biotec, Bergisch Gladbach, Germany).

Flow sorting was performed on a MoFlo™ XDP cytometer (Beckman Coulter GmbH, Krefeld, Germany) at the Institute for Transplantation Diagnostics and Cell Therapeutics (University Hospital Düsseldorf) to isolate tumor cells. Single‐cell sorting utilized a 100 μm nozzle with standard optical configuration (488 nm Laser, 120 mW, detecting GFP through a 529/28 band‐pass filter and a 575/25 nm secondary filter). Cellular debris was excluded via Forward Scatter/Side Scatter (FSC/SSC) gating, while doublets/aggregates were eliminated by using SSC‐width versus SSC‐height (SSC‐W/SSC‐H) to isolate single cells.

Due to the GFP signal in CAM‐derived sample, tumor cells were identified on FL1 (529/28) versus FL2 (575/25) dot blots, where autofluorescent cells formed a diagonal distribution and GFP^+^ events appeared as FL1‐bright populations right‐shifted from this diagonal (Fig. S2A). Specificity was validated using nontumor‐bearing CAM blood controls (*n* = 5), with no GFP^+^ events detected in the defined gate (Fig. S2B). Single GFP^+^ tumor cells were sorted into individual 0.2 mL polymerase chain reaction (PCR) tubes for downstream analyses.

### 2.5 Enrichment of CTCs using CellSearch®, KingFisher® and VyCap®

To validate CTC detection independently of GFP expression, blood samples from CAMs bearing non‐GFP‐expressing CAPAN‐1 xenografts were analyzed using three distinct enrichment/detection platforms.

The FDA‐cleared CellSearch® system employs EpCAM‐dependent immunomagnetic separation (ferrofluids), followed by immunofluorescence staining for 4′,6‐diamidino‐2‐phenylindole (DAPI), cytokeratins (panCK), and CD45. Blood from CAPAN‐1 tumor‐bearing CAMs was collected in CellSearch® tubes, fixed for 24 h using 30 μL of CellSave® preservative reagent (Menarini, Florence, Italy) per 1 mL CAM blood, and adjusted to 8 mL final volume using CellSearch® dilution buffer. CTC enrichment and staining were performed per manufacturer's instructions (CellSearch**®** Circulating Tumor Cell Kit Epithelia—IVD, Menarini).

An alternative EpCAM‐based approach utilized the KingFisher® system (Thermo Fisher Scientific GmbH, Dreieich, Germany) with Dynabeads™ Biotin Binder beads conjugated to anti‐EpCAM antibody (clone VU‐1D9) [23]. CAM blood was fixed in CellSave® tubes for 2 h as described above. For enrichment, 62 μL Dynabeads™ were resuspended in 200 μL binding buffer (PBS + 0.1% bovine serum albumin (BSA) + 2 mm EDTA). The following immunofluorescence antibody panel was used for labeling: AF647‐conjugated anti‐CD45 (clone HI30, 4 μg·mL^−1^, BioLegend; USA); AF488‐conjugated anti‐CK19 (clone A53‐B/A2 at 3.5 μg·mL^−1^; Exbio, Czechia); AF488‐conjugated anti‐panCK (clone C11 at 3.5 μg·mL^−1^; Abcam, UK). Antibodies were diluted in 1× BD Perm/Wash™ (BD Biosciences, San Jose, CA, USA) to 200 μL total volume. Nuclei were counterstained with Hoechst 33342 (2 μg·mL^−1^, Invitrogen, Carlsbad, CA, USA).

For label‐free, size‐based CTC detection, the VyCap® system (VyCAP B.V., Enschede, Netherlands) was employed. Blood collection syringes were preloaded with 50 μL Transfix® reagent (Vacutest Kima, Arzergrande, Italy). Samples were diluted with PBS up to a final volume of 1 mL, fixed for 24 h at room temperature, and processed at the VyCap® laboratory. Filtration occurred at pressures ranging from 25 to 250 mbar. Captured cells were stained with DAPI for nuclear DNA and dual anti‐pan‐cytokeratin antibodies: PE‐conjugated anti‐pan‐cytokeratin antibody (clone CK‐11; 1 μg·mL^−1^; Cell Signaling Technology, Danvers, MA, USA) and eFluor™ 570‐conjugated anti‐pan‐CK antibody (clone AE1/AE3; 1 : 100; eBioscience, San Diego, CA, USA 41–9003‐82).

### 2.6 Whole‐genome amplification and genetic analysis of single CTCs

Individual CTCs isolated from CAM blood by flow sorting underwent single‐cell whole‐genome amplification (WGA) using the Ampli‐1™ WGA Kit (Menarini Silicon Biosystems, Castel Maggiore, Italy) based on Msel adapter‐linker PCR [24, 25]. WGA product quality was assessed via control multiplex PCR [26], samples achieving a genome integrity index (GII) ≥ 3 were considered suitable for downstream analyses [27, 28].

Short‐tandem repeat (STR) profiling was performed at the Institute for Forensic Medicine (University Hospital Düsseldorf) using the PowerPlex® 21 System (Promega GmbH, Walldorf, Germany), with 3 μL of single‐cell WGA product per reaction. STR profiles were analyzed using CLASTR [29] using standard parameters: Tanabe Score, nonempty markers, 60% score filter, minimal eight markers, and maximal 20 results. Next‐generation sequencing (NGS)‐based targeted hotspot mutation analysis interrogated *KRAS* (Exon 2: G12C, G12D, G12V, G13D) and *TP53* (Exon 5: A159V, R175H; Exon 7: R248W; Exon 8: R273H) in single CTCs [6, 30]. For copy number alteration (CNA) detection, quality‐controlled WGA products from single CAM CTCs were converted to Illumina‐compatible libraries using the Ampli1™ LowPass kit (Menarini Silicon Biosystems, Castel Maggiore, Italy) per manufacturer protocol followed by low‐pass whole‐genome sequencing on an Illumina NextSeq platform (Illumina, San Diego, CA, USA).

Sequencing data were processed through: (a) hg19 alignment, (b) CNA inference via quantitative DNA sequencing analysis pipeline (QDNAseq) (500 kb bins), and (c) quality filtering excluding profiles with derivative log ratio spread (DLRS) > 0.35 or interquartile range (IQR) > 0.35 [6, 31]. The final CNA profiles were compared against reference datasets [32, 33].

### 2.7 Experimental design and comparative strategy

To enable biologically meaningful interpretation of growth and dissemination phenotypes, analyses throughout this section were performed using pairwise comparisons within the same tumor entity: CRC (HCT‐116 vs. SW‐620), PDAC (CAPAN‐1 vs. Mia‐PaCa2), and CCA (HuCC‐T1 vs. TFK‐1). This design allows entity‐specific biological differences to be controlled while assessing intrinsic cell line‐specific behaviors.

## 3 Results

### 3.1 Robust tumor engraftment of human GI cancer cell lines on the CAM

**Fig. 1:**
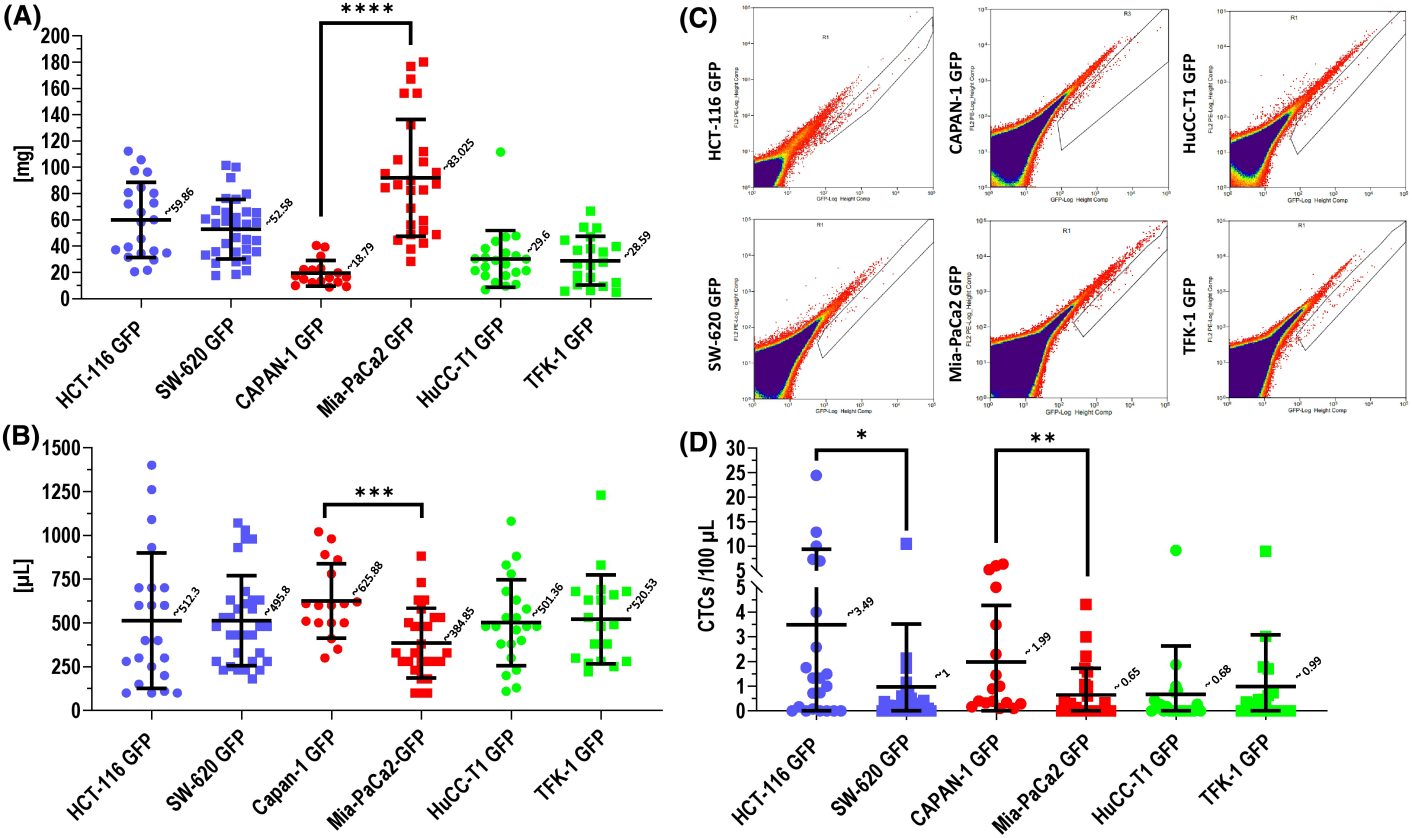
(A) Tumor weights (mg) of six different green fluorescent protein (GFP)‐labeled human gastrointestinal (GI) cancer cell lines xenografted onto the chorioallantoic membrane (CAM) (each dot = one tumor; HCT‐116 GFP: 59.9 ± 28.54 mg, *n* = 22, vs. SW‐620 GFP: 52.6 ± 22.66 mg, *n* = 32, *P* = 0.39, Mann–Whitney *U* test; CAPAN‐1 GFP: 18.8 ± 9.74 mg, *n* = 17, vs. Mia–PaCa2 GFP: 83 ± 44.74 mg, *n* = 27, ****P < 0.0001, Mann–Whitney *U* test; HuCC‐T1 GFP: 29.6 ± 21.58 mg, *n* = 22 vs. TFK‐1 GFP: 28.6 ± 18.45 mg, *n* = 19, *P* = 0.97, Mann–Whitney *U* test). Error bars represent mean ± standard deviation (SD). (B) Blood volume (μL) collected per CAM for each tumor model (each dot = one blood sample; HCT‐116 GFP: 512.3 ± 385.62 μL, *n* = 22, vs. SW‐620 GFP: 495.8 ± 256.31 μL, *n* = 32, *P* = 0.63, Mann–Whitney *U* test; CAPAN‐1 GFP: 628.9 ± 212.25 μL, *n* = 17, vs. Mia–PaCa2 GFP: 384.9 ± 199.18 μL, *n* = 27, ***P < 0.0005, Mann–Whitney *U* test; HuCC‐T1 GFP: 501.36 ± 244.9 μL, *n* = 22 vs. TFK‐1 GFP: 520.33 ± 253.3 μL, *n* = 19, *P* = 0.82, Mann–Whitney *U* test). Error bars represent mean ± standard deviation (SD). (C) Representative flow cytometry plots (GFP^+^ events in R1 gate). (D) GFP^+^ circulating tumor cells (CTCs)/100 μL CAM blood (each dot = one CTC/100 μL value; HCT‐116 GFP: 3.49 ± 5.9 CTCs/100 μL, *n* = 22, vs. SW‐620 GFP: 1 ± 2.55 CTCs/100 μL, *n* = 32, *P = 0.029, Mann–Whitney *U* test; CAPAN‐1 GFP: 1.99 ± 2.28 CTCs/100 μL, *n* = 17, vs. Mia–PaCa2 GFP: 0.65 ± 1.08 CTCs/100 μL, *n* = 27, **P = 0.0032, Mann–Whitney *U* test; HuCC‐T1 GFP: 0.68 ± 1.95 CTCs/100 μL, *n* = 22 vs. TFK‐1 GFP: 0.99 ± 2.1 CTCs/100 μL, *n* = 19, *P* = 0.309, Mann–Whitney *U* test). Error bars represent mean ± standard deviation (SD). No correlation between tumor weight and CTCs/100 μL (HCT‐116 GFP Pearson's *r* = −0.4197, *R* ^2^ = 0.1761. *n* = 22; SW‐620 GFP Pearson's *r* = −0.05577, *R* ^2^ = 0.00311, *n* = 32; CAPAN‐1 GFP: Pearson's *r* = 0.102, *R* ^2^ = 0.0104, *n* = 17; Mia‐PaCa2 GFP Pearson's *r* = 0.18277, *R* ^2^ = 0.03337, *n* = 27; HuCC‐T1 GFP: Pearson's *r* = 0.8113, *R* ^2^ = 0.6582, *n* = 22; TFK‐1 GFP: Pearson's *r* = −0.2693, *R* ^2^ = 0.07251, *n* = 19, see Fig. S4).

All six GFP‐expressing human GI cancer cell lines consistently formed cohesive CAM tumors, confirming robust engraftment (Tables S1, S2 and Fig. S3). Tumor growth patterns varied by tissue origin. CRC cell lines HCT‐116 (59.9 mg, *n* = 22) and SW‐620 (52.6 mg, *n* = 32) grew xenografts of comparable mean weights (*P* > 0.05, Fig. 1A). CCA cell lines exhibited lower overall weight (HuCC‐T1: 29.6 mg, *n* = 22; TFK‐1: 28.6 mg, *n* = 19) relative to CRC. Striking growth disparities emerged among the PDAC cell lines: CAPAN‐1 formed minimal CAM tumors (mean: 18.8 mg, *n* = 17), while Mia‐PaCa2 yielded significantly larger masses (mean: 83.0 mg, *n* = 27; CAPAN‐1 vs. Mia‐PaCa2: *P* < 0.0001, Mann–Whitney *U* test, Fig. 1A).

### 3.2 CTC detection in CAM blood

Blood collected from CAM vasculature prior to tumor harvesting revealed spontaneous release of GFP^+^ CTCs across all six models. Individual blood volumes ranged from 100 to 1400 μL (Fig. 1B, Table S2), with no significant differences among CRC models (HCT‐116 mean volume: 512.3 μL, *n* = 22; SW‐620: 495.8 μL, *n* = 32) or CCA (HuCC‐T1 mean volume: 501.36 μL, *n* = 22; TFK‐1: 520.33 μL, *n* = 19). PDAC cell lines exhibited divergent volumes; Mia‐PaCa2 explants showed low blood volumes (mean: 384.9 μL), while CAPAN‐1 explants reached significantly higher volumes (mean: 628.9 μL; *P* = 0.0005, Mann–Whitney *U*), indicating biological differences beyond tumor growth.

Flow cytometry detected GFP^+^ single CTCs in all six models (Fig. 1C). This EpCAM‐independent approach was chosen to provide a cost‐effective and marker‐independent validation of CTC shedding. To ensure high specificity despite the inherently low frequency of events, gating was strictly defined using nontumor‐bearing controls (negative controls) (Fig. S2B), showing zero false positives across all control samples. To quantify these findings, we defined the *CTC detection rate* as the percentage of eggs where at least one CTC was identified and the *CTC burden* as the concentration of events per 100 μL blood. Detection rates varied: 100% (CAPAN‐1) > 72.2% (HCT‐116) > 59.4% (SW‐620) > 57.9% (TFK‐1) > 48.2% (Mia‐PaCa2) > 45.5% (HuCC‐T1). Mean CTC burden also differed markedly (Fig. 1D): CRC: HCT‐116 (3.49 CTCs/100 μL) > SW‐620 (1 CTC/100 μL; *P* = 0.029, Mann–Whitney *U*); PDAC: CAPAN‐1 (1.99 CTCs/100 μL) > Mia‐PaCa2 (0.65 CTCs/100 μL; *P* = 0.0032, Mann–Whitney *U*); CCA: TFK‐1 (0.99 CTCs/100 μL) > HuCC‐T1 (0.68 CTCs/100 μL). Regression analyses showed no correlation between tumor weight and CTC frequency in any model (Fig. S4). This was particularly evident in the PDAC model, where Mia‐PaCa2 formed significantly larger tumors than CAPAN‐1 but yielded a lower CTC burden.

### 3.3 CAM CTC detection via established enrichment platforms

**Fig. 2:**
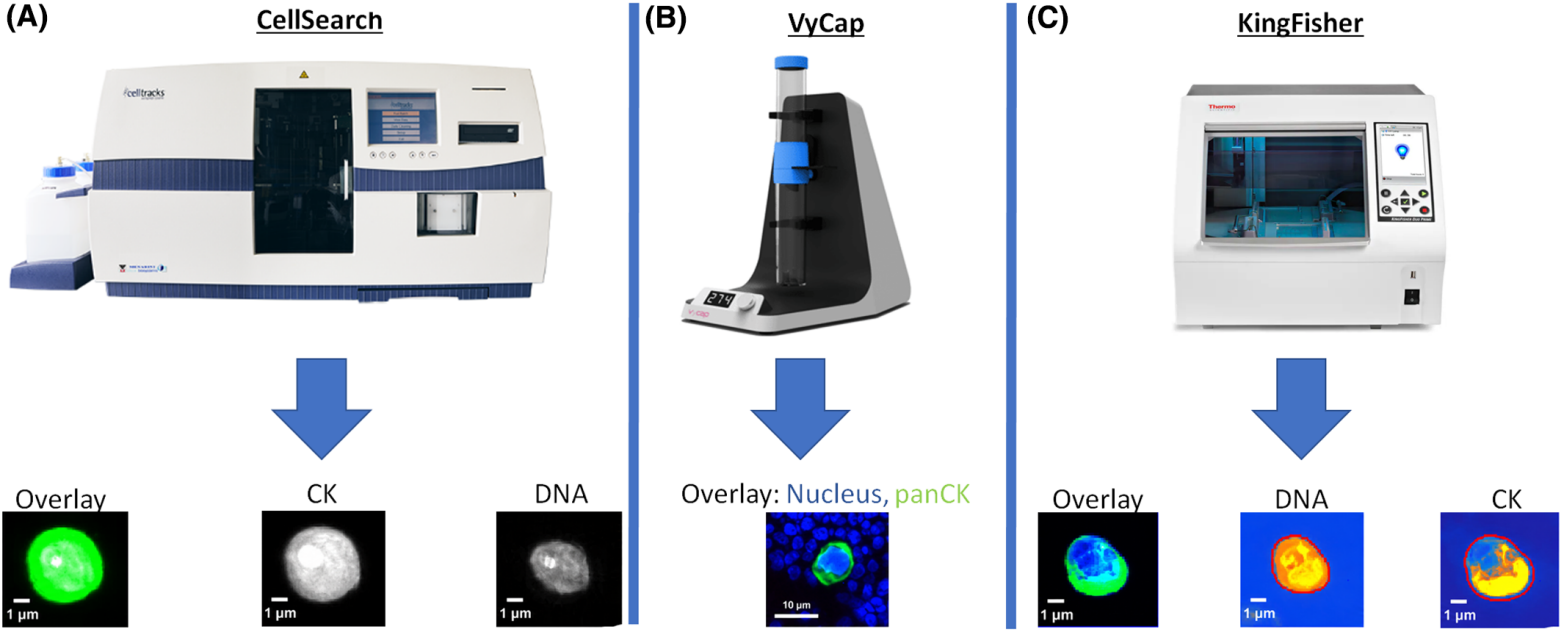
Representative images of circulating tumor cells (CTCs) enriched from pooled CAPAN‐1 chorioallantoic membrane (CAM) blood using (A) CellSearch® (233 CTCs from 13 CAMs); scale bar = 1 μm, (B) VyCap® (*n* = 7 CAMs; total: 16 CTCs; range 1–11 CTCs); scale bar = 10 μm, and (C) KingFisher® (*n* = 9 CAMs; total: 29 CTCs; range 2–13 CTCs); scale bar = 10 μm. Markers: DAPI, 4′,6‐diamidino‐2‐phenylindole (blue); CK/panCK, cytokeratin/pan‐cytokeratin (green/red). (Device illustrations were adapted from the manufacturers' official websites). For CAM data and volume normalization, refer to Table S3.

To validate CTC detection, blood from CAMs bearing non‐GFP‐expressing CAPAN‐1 tumors was analyzed using three commercially available CTC enrichment platforms: CellSearch® (Fig. 2A), VyCap® (Fig. 2B), and KingFisher® (Fig. 2C). All methods identified DAPI^+^/CK^+^ cells with size/morphology consistent with CAPAN‐1 cells (Fig. 2A–C), confirming preserved epithelial marker expression after CAM growth and dissemination.

Using CellSearch®, pooled blood samples from 13 CAPAN‐1 CAMs yielded a total of 233 CTCs in a total CAM blood volume of 5020 μL, corresponding to an average of approximately 4.64 CTCs/100 μL per CAM (Table S3). In contrast, with VyCap® and KingFisher®, the absolute CTC counts per egg showed a stochastic distribution (VyCap®: *n* = 7, total of 16 CTCs, range 0–11; KingFisher®: *n* = 9, total of 29 CTCs, range 0–13), the volume‐normalized CTC concentrations were remarkably consistent between the two platforms (Fig. 2). Analysis revealed a mean CTC burden of 0.65 CTCs/100 μL for VyCap® and 0.69 CTCs/100 μL for KingFisher® (Table S3). This high level of interplatform consistency demonstrates the reliability of CTC detection in the CAM model, with variability remaining within the range reported for conventional murine xenograft models, where substantial short‐term fluctuations in CTC counts have been described [34].

### 3.4 Genetic profiling confirms human origin and cell line identity of isolated CTCs

**Fig. 3:**
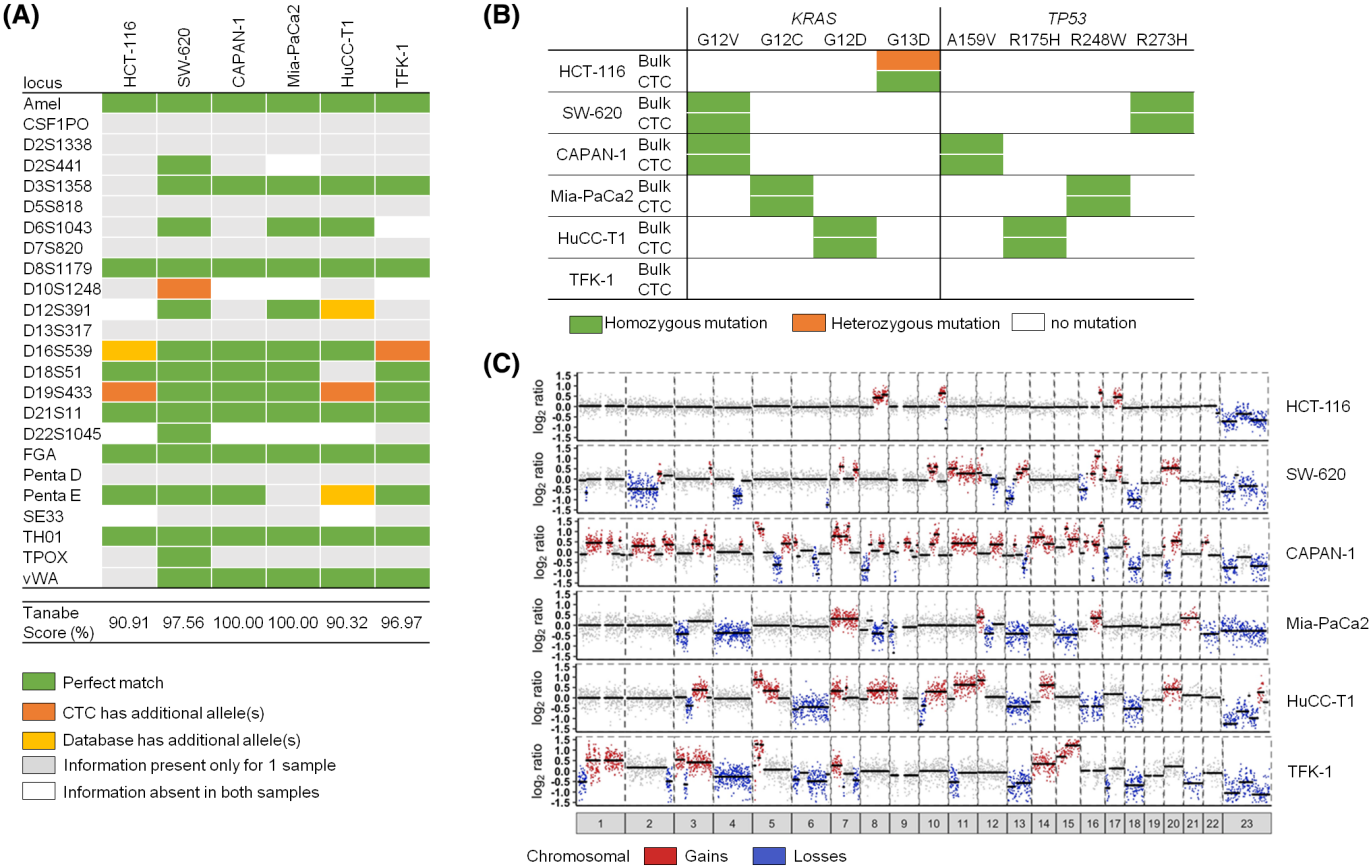
Genomic validation of chorioallantoic membrane (CAM)‐derived circulating tumor cells (CTCs). (A–C) Representative molecular profiles of at least one individual CTC per cell line (A) short‐tandem repear (STR) Tanabe score (%) of single CAM‐derived CTCs isolated from xenografts. (B) *KRAS/TP53* hot spot mutations in bulk DNA vs. single CTCs (homo−/heterozygous). A detailed breakdown of all analyzed CTCs is provided in Table S5. (C) Copy number alteration (CAN) profiles of single CTCs (gains: red, losses: blue; compared to parental lines from [33]). The complete set of all successfully generated CNA profiles is displayed in Fig. S5.

Comprehensive genetic analyses of single GFP^+^ CTCs unequivocally confirmed their tumor origin. Following the stringent quality control criteria (GII ≥ 3 after WGA), only CTCs with high DNA integrity were utilized for downstream applications to ensure the highest data reliability. Although this selection process resulted in a limited number of analyzed single CTCs for some entities, cells were harvested from independent biological replicates to ensure representative sampling (Fig. 3). All analyzed CAM CTCs are shown in Fig. S5.

Short‐Tandem Repeat (STR) profiling authenticated human origin and cell line identity despite partial locus dropouts inherent to MseI‐based single‐cell WGA. CLASTR database analysis yielded unambiguous identifications with Tanabe scores ranging from 90.32% to 100% (Fig. 3A, Table S4), demonstrating near‐perfect concordance of single sorted CTCs with parental cell lines.

Targeted hotspot mutation analysis of *KRAS* (Exon 2: G12C, G12D, G12V, G13D) and *TP53* (Exon 5: A159V, R175H; Exon 7: R248W; Exon 8: R273H) revealed complete concordance between CTCs and bulk parental DNA in 5/6 models (Fig. 3B, Table S5). HCT‐116‐derived CTCs exhibited homozygous *KRAS* mutations versus heterozygous bulk DNA.

Low‐pass whole‐genome sequencing detected characteristic malignant CNAs in all single CTCs (Fig. 3C). High inter‐CTC concordance in CAPAN‐1 and TFK‐1 (Fig. S5) confirmed technical reproducibility. Notably, all genomic profiles remained 100% consistent with the parental lines, even in models where only a small number of high‐quality cells were available for analysis.

Collectively, STR, hotspot mutation, and CNA analyses (Fig. 3A–C) validate CAM‐derived CTCs as molecularly faithful representations of parental CAM tumors, establishing the CAM's technical rigor for dissemination studies.

## 4 Discussion

CRC, PDAC, and CCA remain among the most lethal gastrointestinal malignancies due to their propensity for early systemic dissemination [35, 36, 37, 38, 39, 40, 41, 42, 43]. While CTCs hold prognostic and biological significance, their translational exploitation has been hampered by extreme rarity and technical challenges in single‐cell isolation and characterization [6, 7, 8]. Unlike cell‐free DNA, CTCs provide intact cellular material for comprehensive molecular and functional analyses [13, 14, 15, 44].

Murine models, while invaluable for metastasis research, face limitations in scalability, ethical constraints, and technical feasibility for CTC studies—particularly when investigating early dissemination in small blood volumes [21, 45, 46, 47, 48, 49]. This necessitates alternative *in vivo* platforms that enable accessible, ethically less restricted, and highly parallelized investigation of hematogenous dissemination. Small CAM blood volumes may limit detection of rare events, but the high spontaneous CTC frequency enabled reliable quantification. A major advantage of the CAM assay is its exceptional scalability; it facilitates high‐throughput experimentation due to its cost‐efficiency, minimal space requirements, and the ability to process large cohorts in parallel. This makes the platform uniquely suited for larger scale studies, such as high‐content drug screening programs aimed at identifying compounds that inhibit intravasation or circulatory survival, which would be logistically and ethically impractical in mammalian systems.

We demonstrate that the avian chorioallantoic membrane assay is a robust *in vivo* platform for studying spontaneous CTC formation. Human GI cancer cell lines consistently formed tumors and released viable CTCs into the circulation, with detection rates ranging from 45.5% to 100% across entities. Crucially, CTC isolation was validated using three clinical platforms (CellSearch® [50, 51], KingFisher® [23] and VyCap® [52]), confirming retention of epithelial markers (CK^+^/DAPI^+^ cells) independent of GFP labeling. This multiplatform concordance underscores the biological relevance of CAM‐derived CTCs.

However, we observed a discrepancy between the blood volume‐normalized CTC concentrations (Fig. 1, Table S3) obtained via flow cytometry and those detected by enrichment platforms. While flow cytometry detected 1.99 CTCs/100 μL for CAPAN‐1, the detected CTC burden using VyCap® and KingFisher® was comparatively lower (mean: 0.65 and 0.69 CTCs/100 μL, respectively). This is likely attributable to the multi‐step nature of clinical enrichment protocols, which—unlike direct flow cytometry analysis—rely on specific immuno‐magnetic or size‐based filtration steps. Such processes inherently involve a degree of cell loss. Furthermore, the dependence on antibodies (e.g., EpCAM‐based capture) and the mechanical stress during sample processing may contribute to lower detection rates. Intriguingly, the CellSearch® system detected the highest CTC burden, with a mean of 4.64 CTCs/100 μL, outperforming both enrichment platforms and the flow cytometry sorting. This superior performance highlights the high efficiency of the EpCAM‐targeted ferrofluid technology utilized by CellSearch®, which minimizes cell loss and mechanical shear stress often associated with the filtration membranes of VyCap® or the bead‐handling steps of the KingFisher® protocol. Furthermore, while flow cytometry (1.99 CTCs/100 μL) is limited by the analysis of relatively small sample aliquots—rendering it susceptible to sampling errors or underestimation in clumped cell suspensions—the CellSearch® workflow screens the entire adjusted sample volume. Nevertheless, the consistency of normalized concentrations across the above technologies demonstrates that the CAM model provides a robust and reproducible platform for CTC research. This high level of interplatform consistency demonstrates the reliability of CTC detection in the CAM model, with variability remaining within the range reported for conventional murine xenograft models, where substantial short‐term fluctuations in CTC counts have been described [34].

Although flow cytometry enabled GFP‐based detection of spontaneously shed tumor cells, this approach has inherent limitations for the analysis of rare cellular events and may be influenced by background autofluorescence. To minimize false‐positive events, gating was established using nontumor‐bearing CAM blood controls, in which no GFP‐positive events were detected. Moreover, the dissemination patterns observed by flow cytometry were independently confirmed using three established CTC detection platforms based on distinct enrichment principles, supporting that the observed findings are not specific to the GFP‐based detection strategy.

Comprehensive single‐cell genomic characterization of CAM‐derived single CTCs—including STR profiling, targeted hotspot mutation analysis (*KRAS/TP53*), and low‐pass whole‐genome sequencing—confirmed human origin and genetic fidelity to parental cell lines. The homozygous *KRAS* mutation observed in HCT‐116‐derived CTCs likely reflects artifacts of single‐cell genomics rather than biological divergence. While the use of established cancer cell lines inherently limits the assessment of intertumoral heterogeneity, the applied single‐cell approaches are fully compatible with more heterogeneous systems, including patient‐derived materials.

Although the number of genetically characterized single CTCs in the present study was limited, the results support our aim to establish the technical feasibility of single‐cell isolation and downstream molecular characterization of spontaneously shed CAM‐derived CTCs. The high concordance observed between CTCs and their parental cell lines supports the robustness of the workflow. Yet, larger confirmatory studies including substantially higher numbers of single CTCs and additional tumor models may be required to establish this approach for broader applications in CTC research.

The CAM model presents inherent limitations requiring consideration. Differences in avian circulatory physiology and the absence of adaptive immunity preclude direct extrapolation of CTC frequencies or prognostic implications to human contexts. Consequently, CAM‐derived CTCs should not be regarded as quantitative surrogates for patient‐derived counterparts. Rather, the strength of the model lies in providing mechanistic access to early dissemination events—including intravasation and circulatory survival—under controlled *in vivo* conditions. Furthermore, the CAM assay offers substantial advantages as a complementary *in vivo* model. Its scalability, cost‐efficiency, technical accessibility, and alignment with 3R principles enable experimental designs impractical in mammalian systems. The platform enables rapid parallel experimentation with minimal material requirements, permitting comparative analyses across tumor entities or conditions. Critically, reproducible CTC detection across CRC, PDAC, and CCA models underscores its broad applicability for studying early hematogenous dissemination.

Importantly, the dissemination phenotypes observed in the CAM assay are consistent with prior findings from mammalian *in vivo* models. Consistent with murine models, HCT‐116 shed more CTCs than SW‐620, and CAPAN‐1 showed higher CTC frequencies than Mia‐PaCa2 despite originating from smaller tumors [53, 54]. The divergent phenotypes of Mia‐PaCa2 and CAPAN‐1 we report here may be explained by their distinct biological characteristics. While Mia‐PaCa2 is known for its high proliferative capacity [54] and its ability to form large primary masses, its metastatic potential may be limited by a less efficient intravasation process [55]. In contrast, CAPAN‐1 cells, which formed smaller primary tumors, are reported to exhibit a more invasive signaling profile that favors early systemic shedding [54, 55]. Regression analyses showed no correlation between tumor weight and CTC frequency, indicating that CAM tumor dissemination occurs independently of bulk tumor growth. Intravasation critically depends on the development and functionality of the intratumoral vasculature and the local tumor microenvironment [56]. Rapid tumor expansion may outpace the establishment of a sufficiently functional vascular network, potentially resulting in necrotic tumor regions with reduced access to the circulation [57]. This may reflect tumor size–associated alterations in local vascular architecture and perfusion, potentially limiting effective blood recovery in larger CAM tumors [58]. The concordance between murine CCA data and our findings across three GI cancer entities supports the CAM's capacity to recapitulate intrinsic cancer‐type‐specific dissemination patterns [59].

Beyond modeling spontaneous CTC generation, the CAM assay holds significant translational potential [60, 61]. The primary prerequisite for such applications is the successful engraftment and vascularization of patient‐derived organoids (PDOs) or primary tumor fragments on the CAM. This would allow for the functional stratification of a patient's individual tumor based on its ‘dissemination competence’ in an *in vivo* environment. Regarding the technical feasibility of detecting patient‐derived material, our pipeline may enable detection of patient‐derived CTCs and controlled *in vivo* testing of antidissemination therapies, thereby linking liquid biopsy analyses with functional assessments of early metastatic progression.

## 5 Conclusion

This study establishes the avian CAM assay as a biologically relevant, experimentally accessible, and genetically traceable *in vivo* model for isolating and investigating spontaneous CTC formation in gastrointestinal cancers. By explicitly focusing on early hematogenous dissemination—rather than metastatic colonization—the CAM assay complements existing mammalian models and bridges the methodological gap between simplified *in vitro* assays (e.g., proliferation, migration, and invasion assays) and complex murine *in vivo* models. As the first model enabling reproducible, scalable isolation and comprehensive molecular validation of spontaneously shed CTCs across multiple GI cancer entities, it represents a transformative tool for advancing mechanistic studies of tumor cell dissemination and accelerating the development of antimetastatic therapies.

## Conflict of interest

The authors have no conflicts of interest to declare.

## Author contributions

DR, GF, and MS were involved in data curation. DR, KCA, and MS were involved in formal analysis. GF and NHS were involved in funding acquisition. DR, JG, CB, NG, KCA, CÖ, SHL, AF, and HN were involved in investigation. DR and GF were involved in methodology. GF was involved in project administration. SS, CR, and WTK were involved in resources. GF, NHS, and RPLN were involved in supervision. DR, RPLN, EE, and GF were involved in visualization. DR, NHS, and GF were involved in writing.

## Supporting information


**Table S1.** Engraftment success of gastrointestinal cancer cell lines on the chorioallantoic membrane.
**Table S2.** Raw data of CAM tumor weights, blood volumes, and circulating tumor cell counts for colorectal, pancreatic, and cholangiocarcinoma models.
**Table S3.** Quantification of circulating tumor cells using CellSearch®, VyCap®, and KingFisher® platforms.
**Table S4.** STR profiling confirms the identity of CAM‐derived circulating tumor cells.
**Table S5.** KRAS and TP53 hotspot mutation analysis of CAM‐derived circulating tumor cells.
**Fig. S1.** Preparation of the avian chorioallantoic membrane (CAM).
**Fig. S2.** Flow cytometric gating strategy for GFP‐positive circulating tumor cells (CTCs) in CAM blood.
**Fig. S3.** Representative *ex vivo* and *in vivo* images of CAM tumors generated by human gastrointestinal cancer cell lines.
**Fig. S4.** Correlation between tumor weight and circulating tumor cell burden in colorectal, pancreatic, and cholangiocarcinoma CAM models.
**Fig. S5.** Copy number alteration (CNA) profiles of all analyzed CAM‐derived circulating tumor cells.

## Data Availability

The datasets generated and analyzed are available from the corresponding author upon reasonable request. All data supporting the findings of this study will be provided in a de‐identified format to qualified researchers following completion of a material transfer agreement.
