# Supplementary material for: Bridging the gap: a genetically validated avian chorioallantoic membrane platform for investigation of spontaneous circulating tumor cells
Source: Mol Oncol. 2026 Sep 18;20(11):2611–23. doi: 10.1002/1878-0261.70336 (PMC13587589; doi:10.1002/1878-0261.70336)
Supplement: Supplementary file 1 [file MOL2-20-2611-s001.docx]

**Supplementary**

**CAM Dropping**

 ***Supplementary Fig. 1****:* ***Preparation of avian chorioallantoic membrane (CAM)****. 1. Incubation of eggs for 10 days at 37.5°C and 48% humidity. 2. Optical control for fertilization and vascularization. 3. Marking of well vascularized area and piercing into the air sac (blunt end). 4. Breakthrough and check of removed egg shell. 5. Repositioning of the air sac. 6. Removal of calcareous shell with a rotating blade. 7. Remove egg shell to open a window. 8. Inoculation and closure.*

*Supplementary Fig. 2: Flow cytometric gating strategy for green fluorescent protein (GFP)-positive circulating tumor cells CTCs in CAM blood. (A) Representative gating based on FL1 (529/28) versus FL2 (575/25) fluorescence followed by exclusion of debris and doublets. Auto-fluorescent GFP-negative cells form a diagonal distribution. (B) Representative negative control derived from blood of non-tumor-bearing CAMs, demonstrating absence of events within the GFP-positive gate (n = 5).*

|  | CAMs with no visible tumor | Dead CAMs following inoculation | CAMs with tumor |
| --- | --- | --- | --- |
| HCT-116 GFP | 1 (3.125%) | 9 (28.125%) | 22 (68.75%) |
| SW-620 GFP | 5 (12.5%) | 3 (7.5%) | 32 (80%) |
| CAPAN-1 GFP | 1 (4.55%) | 4 (18.18%) | 17 (77.27%) |
| Mia-PaCa2 GFP | 1 (2.86%) | 7 (20%) | 27 (77.14%) |
| HuCC-T1 GFP | 2 (8%) | 1 (4%) | 22 (88%) |
| TFK-1 GFP | 3 (13.04%) | 1 (4.35%) | 19 (82.61%) |

***Supplementary Table 1****: Success rate of xenografted gastrointestinal (GI) cancer cell lines onto the chorioallantoic membrane (CAM)*

| **HCT-116 GFP** | | | | **SW-620 GFP** | | | | **CAPAN-1 GFP** | | | | **Mia-PaCa2 GFP** | | | | **HuCC-T1 GFP** | | | | **TFK-1 GFP** | | | |
| --- | --- | --- | --- | --- | --- | --- | --- | --- | --- | --- | --- | --- | --- | --- | --- | --- | --- | --- | --- | --- | --- | --- | --- |
| **number of CAMs** | **tumor weight in mg** | **blood volume in µL** | **number of CTCs** | **number of CAMs** | **tumor weight in mg** | **blood volume in µL** | **number of CTCs** | **number of CAMs** | **tumor weight in mg** | **blood volume in µL** | **number of CTCs** | **number of CAMs** | **tumor weight in mg** | **blood volume in µL** | **number of CTCs** | **number of CAMs** | **tumor weight in mg** | **blood volume in µL** | **number of CTCs** | **number of CAMs** | **tumor weight in mg** | **blood volume in µL** | **number of CTCs** |
| 1 | 72,8 | 150 | 11 | 1 | 65,6 | 1030 | 1 | 1 | 23,1 | 600 | 1 | 1 | 177 | 180 | 0 | 1 | 38,2 | 110 | 0 | 1 | 35,4 | 490 | 0 |
| 2 | 39,3 | 100 | 10 | 2 | 100 | 530 | 2 | 2 | 22,3 | 500 | 5 | 2 | 156 | 100 | 3 | 2 | 34,8 | 400 | 1 | 2 | 5 | 690 | 62 |
| 3 | 37,2 | 600 | 8 | 3 | 28,1 | 430 | 0 | 3 | 21,8 | 600 | 2 | 3 | 156 | 280 | 0 | 3 | 9,5 | 480 | 4 | 3 | 6,3 | 220 | 1 |
| 4 | 96,4 | 700 | 0 | 4 | 75,6 | 230 | 1 | 4 | 40,5 | 510 | 2 | 4 | 86,6 | 480 | 4 | 4 | 11 | 200 | 2 | 4 | 5,9 | 280 | 5 |
| 5 | 80,7 | 250 | 0 | 5 | 61,7 | 230 | 24 | 5 | 9,3 | 890 | 8 | 5 | 95,9 | 730 | 1 | 5 | 30,4 | 530 | 0 | 5 | 12,3 | 380 | 0 |
| 6 | 31,6 | 700 | 171 | 6 | 58,2 | 630 | 5 | 6 | 32,4 | 410 | 26 | 6 | 112 | 630 | 14 | 6 | 30,2 | 480 | 9 | 6 | 25,2 | 680 | 2 |
| 7 | 29,4 | 930 | 24 | 7 | 21,4 | 280 | 6 | 7 | 19,7 | 610 | 2 | 7 | 95,1 | 630 | 10 | 7 | 26,3 | 380 | 1 | 7 | 54,4 | 270 | 2 |
| 8 | 36,3 | 1090 | 8 | 8 | 35,6 | 180 | 19 | 8 | 39,3 | 860 | 30 | 8 | 87,1 | 880 | 3 | 8 | 12,3 | 680 | 0 | 8 | 66,6 | 250 | 0 |
| 9 | 21,6 | 600 | 77 | 9 | 56,7 | 250 | 0 | 9 | 9,9 | 350 | 1 | 9 | 180 | 280 | 0 | 9 | 6,9 | 530 | 0 | 9 | 27,7 | 680 | 0 |
| 10 | 45,5 | 700 | 0 | 10 | 18,5 | 230 | 0 | 10 | 12,2 | 780 | 39 | 10 | 106 | 580 | 25 | 10 | 46,9 | 300 | 0 | 10 | 46,9 | 300 | 0 |
| 11 | 84,6 | 110 | 0 | 11 | 67,3 | 930 | 0 | 11 | 16,2 | 500 | 2 | 11 | 90 | 480 | 0 | 11 | 112 | 230 | 21 | 11 | 10,7 | 660 | 2 |
| 12 | 112 | 1260 | 1 | 12 | 17,6 | 480 | 0 | 12 | 17,4 | 610 | 14 | 12 | 82,5 | 380 | 0 | 12 | 32,7 | 130 | 1 | 12 | 16,1 | 480 | 3 |
| 13 | 20,6 | 600 | 1 | 13 | 29,5 | 980 | 1 | 13 | 13,4 | 620 | 9 | 13 | 48,8 | 530 | 0 | 13 | 17,6 | 1080 | 0 | 13 | 39,7 | 630 | 0 |
| 14 | 60 | 400 | 0 | 14 | 27 | 280 | 0 | 14 | 14,9 | 500 | 26 | 14 | 42,3 | 280 | 0 | 14 | 21,4 | 480 | 0 | 14 | 17,6 | 380 | 0 |
| 15 | 34,8 | 100 | 7 | 15 | 54,8 | 430 | 5 | 15 | 12,7 | 300 | 18 | 15 | 59,7 | 330 | 0 | 15 | 43,7 | 880 | 1 | 15 | 44,4 | 630 | 3 |
| 16 | 72 | 200 | 3 | 16 | 66,1 | 980 | 0 | 16 | 16,4 | 980 | 1 | 16 | 52,2 | 100 | 0 | 16 | 47,8 | 460 | 2 | 16 | 53,8 | 530 | 16 |
| 17 | 65,8 | 300 | 12 | 17 | 35,8 | 580 | 3 | 17 | 8,8 | 1020 | 1 | 17 | 56,1 | 530 | 0 | 17 | 24,2 | 630 | 0 | 17 | 36,7 | 1230 | 21 |
| 18 | 58,2 | 300 | 4 | 18 | 46,4 | 230 | 4 |  |  |  |  | 18 | 132 | 480 | 0 | 18 | 21,4 | 830 | 0 | 18 | 26,3 | 830 | 3 |
| 19 | 34,5 | 100 | 1 | 19 | 42 | 580 | 3 |  |  |  |  | 19 | 104 | 330 | 1 | 19 | 20,1 | 480 | 0 | 19 | 38,1 | 280 | 0 |
| 20 | 106 | 280 | 2 | 20 | 65,7 | 680 | 0 |  |  |  |  | 20 | 167 | 330 | 2 | 20 | 17,5 | 580 | 1 |  |  |  |  |
| 21 | 97,5 | 400 | 7 | 21 | 58,9 | 530 | 0 |  |  |  |  | 21 | 44,5 | 230 | 4 | 21 | 29,2 | 780 | 0 |  |  |  |  |
| 22 | 80,2 | 1400 | 0 | 22 | 57,4 | 480 | 1 |  |  |  |  | 22 | 82,6 | 280 | 3 | 22 | 31,3 | 380 | 0 |  |  |  |  |
|  |  |  |  | 23 | 60,8 | 630 | 5 |  |  |  |  | 23 | 68,9 | 100 | 1 |  |  |  |  |  |  |  |  |
|  |  |  |  | 24 | 79,7 | 330 | 2 |  |  |  |  | 24 | 28,3 | 500 | 0 |  |  |  |  |  |  |  |  |
|  |  |  |  | 25 | 33,1 | 630 | 1 |  |  |  |  | 25 | 37,8 | 180 | 0 |  |  |  |  |  |  |  |  |
|  |  |  |  | 26 | 35,4 | 610 | 0 |  |  |  |  | 26 | 48,7 | 280 | 0 |  |  |  |  |  |  |  |  |
|  |  |  |  | 27 | 44,1 | 430 | 1 |  |  |  |  | 27 | 84,4 | 280 | 1 |  |  |  |  |  |  |  |  |
|  |  |  |  | 28 | 76,1 | 330 | 1 |  |  |  |  |  |  |  |  |  |  |  |  |  |  |  |  |
|  |  |  |  | 29 | 101 | 1070 | 1 |  |  |  |  |  |  |  |  |  |  |  |  |  |  |  |  |
|  |  |  |  | 30 | 45 | 430 | 0 |  |  |  |  |  |  |  |  |  |  |  |  |  |  |  |  |
|  |  |  |  | 31 | 37,8 | 480 | 0 |  |  |  |  |  |  |  |  |  |  |  |  |  |  |  |  |
|  |  |  |  | 32 | 92,1 | 280 | 0 |  |  |  |  |  |  |  |  |  |  |  |  |  |  |  |  |

***Supplementary Table 2****: Chorioallantoic membrane (CAM) tumor weights, blood volumes, and number of detected circulating tumor cells (CTCs) of colorectal cancer (CRC) (blue), pancreatic ductal adenocarcinoma (PDAC) (red) and cholangiocarcinoma (CCA) (green) cell lines xenografted onto the CAM. Table shows the raw data of each CAM.*

5 mm

5 mm

5 mm

5 mm

5 mm

5 mm

5 mm

5 mm

5 mm

5 mm

5 mm

5 mm

5 mm

5 mm

5 mm

5 mm

5 mm

5 mm

*Supplementary Fig. 3: Chorioallantoic membrane (CAM) tumors ex vivo following dissection (A.1, B.1, C.1, D.1, E.1 & F.1) and in-vivo on the CAM before excision (A.2, B.2, C.2, D.2, E.2 & F.2). Blue arrows indicate CAM tumors. One square represents 1 mm side length.*

*Supplementary Fig. 4: Correlation between circulating tumor cells (CTCs)/100 µL and tumor weight of (A) Colorectal cancer (CRC) (HCT-116 GFP, n = 22; SW-620 GFP, n = 32), (B) Pancreatic ductal adenocarcinoma (PDAC) (CAPAN-1 GFP, n = 17; Mia-PaCa2 GFP, n = 27), and (C) Cholangiocarcinoma (CCA) (HuCC-T1 GFP, n = 22; TFK-1 GFP, n = 19) CTCs and corresponding chorioallantoic membrane (CAM) tumors. The number of CTCs/100 µL did not correlate with tumor weight. Statistical significance was determined using a two-tailed Pearson correlation test. Pearson coefficient r and R² are indicated in the respective panels.*

| **CellSearch® (pooled data)** | | | | **VyCAP® (individual data)** | | | | **KingFisher® (individual data)** | | | |
| --- | --- | --- | --- | --- | --- | --- | --- | --- | --- | --- | --- |
| CAM | CTCs | Pooled Blood volume in µL | CTCs/100 µL | CAM | CTCs | Blood volume in µL | CTCs/100 µL | CAM | CTCs | Blood volume in µL | CTCs/100 µL |
| n = 13 | 233 | 5020 | 4.64 | 1 | 0 | 500 | 0 | 1 | 7 | 550 | 1.27 |
|  |  |  |  | 2 | 0 | 300 | 0 | 2 | 0 | 800 | 0 |
|  |  |  |  | 3 | 0 | 450 | 0 | 3 | 0 | 600 | 0 |
|  |  |  |  | 4 | 1 | 500 | 0.2 | 4 | 13 | 400 | 3.25 |
|  |  |  |  | 5 | 4 | 250 | 1.6 | 5 | 2 | 650 | 0.31 |
|  |  |  |  | 6 | 11 | 400 | 2.75 | 6 | 0 | 450 | 0 |
|  |  |  |  | 7 | 0 | 500 | 0 | 7 | 0 | 550 | 0 |
|  |  |  |  |  |  |  |  | 8 | 7 | 550 | 1.27 |
|  |  |  |  |  |  |  |  | 9 | 0 | 400 | 0 |

***Supplementary Table 3****:* ***Circulating tumor cell (CTC) quantification and volume-normalization for CellSearch^®^ VyCap^®^ and KingFisher^®^ systems.*** *Counts (pooled data for CellSearch^®^, individual data for VyCAP^®^ and KingFisher^®^) of CTCs isolated from CAPAN-1 bearing chorioallantoic membrane (CAMs). The table displays absolute CTC counts per CAM, the respective blood volumes collected, and the calculated CTC concentration per 100 µL (CTC burden) for both enrichment platforms.*

|  | **HCT-116** | | **SW-620** | | **CAPAN-1** | | **Mia-PaCa2** | | **HuCC-T1** | | **TFK-1** | |
| --- | --- | --- | --- | --- | --- | --- | --- | --- | --- | --- | --- | --- |
| **Locus ID** | CTC | CLASTR | CTC | CLASTR | CTC | CLASTR | CTC | CLASTR | CTC | CLASTR | CTC | CLASTR |
|  |  |  |  |  |  |  |  |  |  |  |  |  |
| Amel | X | X | X | X | X | X | X | X | XY | XY | XY | XY |
| CSF1PO |  | 7,9,10,11 |  | 13,14 |  | 11 |  | 10 |  | 11,12 |  | 10 |
| D2S1338 |  | 16 |  | 17,24 |  | 20,24 |  | 25 |  | 17,18 |  | 18,22 |
| D2S441 |  |  | 10,15 | 10,15 | 10,14 |  |  |  | 10,11 |  | 10,11 |  |
| D3S1358 |  | 12,18,19 | 16 | 16 | 15 | 15 | 16 | 16 | 15 | 15 | 17,18 | 17,18 |
| D5S818 |  | 10,11 |  | 13 |  | 11 |  | 12,13 |  | 12,13 |  | 9,12 |
| D6S1043 | 13,14 |  | 11,12 | 11,12 | 21 |  | 12 | 12 | 13 | 13 |  |  |
| D7S820 |  | 11,12 |  | 8,9 |  | 10,11 |  | 12 |  | 10,11 |  | 10 |
| D8S1179 | 12,14 | 12,14 | 13 | 13 | 14,16 | 14,16 | 16 | 16 | 10 | 10 | 13,14 | 13,14 |
| D10S1248 | 14,15 |  | 12,13 | 13 |  |  |  |  | 13 |  |  |  |
| D12S391 |  |  | 17 | 17 | 23 |  | 19 | 19 | 20 | 18,20 | 16,17 |  |
| D13S317 |  | 10,12 |  | 12 |  | 9 |  | 12,13 |  | 11,13 |  | 14 |
| D16S539 | 13,14 | 11,12,13,14 | 9,13 | 9,13 | 13,14 | 13,14 | 13,10 | 10,13 | 11,12 | 11,12 | 9,11 | 9 |
| D18S51 | 16,17 | 16,17 | 13 | 13 | 12 | 12 | 12 | 12 |  | 13 | 16 | 16 |
| D19S433 | 12,13 | 12 | 13 | 13 | 14,15 | 14,15 | 15 | 15 | 12,13 | 13 | 14,15 | 14,15 |
| D21S11 | 29,30 | 29,30 | 30,30.2 | 30,30.2 | 28,30 | 28,30 | 29,31.2 | 29,31.2 | 31 | 31 | 30 | 30 |
| D22S1045 |  |  | 16 | 16 |  |  |  |  |  |  | 16,17 |  |
| FGA | 18,23 | 18,23 | 24 | 24 | 24 | 24 | 22 | 22 | 20,23 | 20,23 | 20,23 | 20,23 |
| Penta D |  | 9,13 |  | 9,15 |  | 9,13 |  | 12,16 |  | 10 |  | 13,14 |
| Penta E | 13,14 | 13,14 | 10 | 10 | 10,12 | 10,12 |  | 13,18 | 18 | 15,18 | 5,18 | 5,18 |
| SE33 |  |  | 19.2,30.2 |  | 18 |  | 16 |  |  |  | 23.2,24.2 |  |
| TH01 | 8,9 | 8,9 | 8 | 8 | 6 | 6 | 9,10 | 9,10 | 7,10 | 7,10 | 6 | 6 |
| TPOX |  | 8 | 11 | 11 |  | 8,11 |  | 9 |  | 8 |  | 8 |
| vWA |  | 17 | 16 | 16 | 16 | 16 | 15 | 15 | 18 | 18 | 14,17 | 14,17 |
|  |  |  |  |  |  |  |  |  |  |  |  |  |
| **Tanabe Score** | 90.91% | | 97.56% | | 100.00% | | 100.00% | | 90.32% | | 96.97% | |

***Supplementary Table 4****: Short tandem repeat (STR) analysis of colorectal cancer (CRC), pancreatic ductal adenocarcinoma (PDAC), and cholangiocarcinoma (CCA) single chorioallantoic membrane (CAM) circulating tumor cells (CTCs). The STR results (CTC) and the proposed cell line of origin (CLASTR) show very high concordance, resulting in Tanabe Scores >90% in all cases.*

|  |  | **Mutation** |  |  |  |
| --- | --- | --- | --- | --- | --- |
|  | **Sample** | ***KRAS*** |  | ***TP53*** |  |
| **HCT-116** | **cell culture DNA** | **G13D** | **heterozygous** | **No mutation detected** | |
|  | **CTC 1** | **G13D** | **homozygous** | **No mutation detected** | |
|  | **CTC 2** | **G13D** | **homozygous** | **No mutation detected** | |
|  | **CTC 3** | **G13D** | **homozygous** | **No mutation detected** | |
|  | **CTC 4** | **G13D** | **homozygous** | **No mutation detected** | |
| **SW-620** | **cell culture DNA** | **G12V** | **homozygous** | **R273H** | **homozygous** |
|  | **CTC 1** | **G12V** | **homozygous** | **R273H** | **homozygous** |
| **CAPAN-1** | **cell culture DNA** | **G12V** | **homozygous** | **A159V** | **homozygous** |
|  | **CTC 1** | **G12V** | **homozygous** | **A159V** | **homozygous** |
|  | **CTC 2** | **G12V** | **homozygous** | **A159V** | **homozygous** |
| **Mia-PaCa2** | **cell culture DNA** | **G12C** | **homozygous** | **R248W** | **homozygous** |
|  | **CTC 1** | **G12C** | **homozygous** | **R248W** | **homozygous** |
| **HuCC-T1** | **cell culture DNA** | **G12D** | **homozygous** | **R175H** | **homozygous** |
|  | **CTC 1** | **G12D** | **homozygous** | **R175H** | **homozygous** |
| **TFK-1** | **cell culture DNA** | **No mutation detected** | | **No mutation detected** | |
|  | **CTC 1** | **No mutation detected** | | **No mutation detected** | |
|  | **CTC 2** | **No mutation detected** | | **No mutation detected** | |

***Supplementary Table 5****:* ***Specific Hotspot mutation analysis in KRAS and TP53*** *of colorectal cancer (CRC), pancreatic ductal adenocarcinoma (PDAC), and cholangiocarcinoma (CCA) single chorioallantoic membrane (CAM) circulating tumor cell (CTC) whole genome amplification (WGA) products (n = 11; HCT-116: n = 4, SW-620: n = 1, CAPAN-1: n = 2, Mia-PaCa2: n = 1, HuCC-T1: n = 1, TFK-1: n = 2) and cell culture DNA was performed as described previously (1, 2).*

*Supplementary Fig. 5: Copy number alteration (CNA) profiles of all analyzed chorioallantoic membrane (CAM) circulating tumor cells (CTCs) (*n = 9; HCT-116: n = 2; SW-620: n = 1; CAPAN-1: n = 2; Mia-PaCa2: n = 1; HuCC-T1: n = 1; TFK-1: n = 2*). Gains are marked in red, losses are marked in blue.*

**References**

1.	Stoecklein NH, Fluegen G, Guglielmi R, Neves RPL, Hackert T, Birgin E, et al. Ultra-sensitive CTC-based liquid biopsy for pancreatic cancer enabled by large blood volume analysis. Mol Cancer. 2023;22(1):181.

2.	Franken A, Honisch E, Reinhardt F, Meier-Stiegen F, Yang L, Jaschinski S, et al. Detection of ESR1 Mutations in Single Circulating Tumor Cells on Estrogen Deprivation Therapy but Not in Primary Tumors from Metastatic Luminal Breast Cancer Patients. J Mol Diagn. 2020;22(1):111-21.
